# Foliar Application or Seed Priming of Cholic Acid-Glycine Conjugates can Mitigate/Prevent the Rice Bacterial Leaf Blight Disease *via* Activating Plant Defense Genes

**DOI:** 10.3389/fpls.2021.746912

**Published:** 2021-09-24

**Authors:** Garima Pal, Devashish Mehta, Saurabh Singh, Kalai Magal, Siddhi Gupta, Gopaljee Jha, Avinash Bajaj, Vemanna S. Ramu

**Affiliations:** ^1^Laboratory of Plant Functional Genomics, Regional Centre for Biotechnology, NCR Biotech Science Cluster, Faridabad, India; ^2^Laboratory of Nanotechnology and Chemical Biology, Regional Centre for Biotechnology, NCR Biotech Science Cluster, Faridabad, India; ^3^Laboratory of Plant Microbe Interactions, National Institute of Plant Genome Research, Aruna Asaf Ali Marg, New Delhi, India

**Keywords:** crop protection, cholic acid, bacterial blight, *Xanthomonas*, sheath blight, *Rhizoctonia solani*

## Abstract

*Xanthomonas Oryzae* pv. *oryzae* (*Xoo*) causes bacterial blight and *Rhizoctonia solani (R. solani)* causes sheath blight in rice accounting for >75% of crop losses. Therefore, there is an urgent need to develop strategies for the mitigation of these pathogen infections. In this study, we report the antimicrobial efficacy of Cholic Acid-Glycine Conjugates (CAGCs) against *Xoo and R. solani*. We show that CAGC C6 is a broad-spectrum antimicrobial and is also able to degrade biofilms. The application of C6 did not hamper plant growth and showed minimal effect on the plant cell membranes. Exogenous application of C6 on pre-infection or post-infection of Xoo on rice susceptible genotype *Taichung native* (TN1) can mitigate the bacterial load and improve resistance through upregulation of plant defense genes. We further demonstrate that C6 can induce plant defense responses when seeds were primed with C6 CAGC. Therefore, this study demonstrates the potential of CAGCs as effective antimicrobials for crop protection that can be further explored for field applications.

## Introduction

Sessile rice plants are always vulnerable to the pathogens, such as bacteria, fungi, and viruses that can lead to >25% of crop losses. Rice bacterial blight caused by *Xanthomonas oryzae* pv. *oryzae* (*Xoo*) is one of the devastating diseases that can severely cause yield losses depending on the variety, growth stage, and environmental conditions (Liu et al., 2014; Varshney et al., 2019). *Xoo* is a Gram-negative bacterium that enters into the rice plants through hydathodes or wounds of leaf-tip. *Xoo* infection causes water-soaked spots at leaf tips and margins that later become chlorotic and necrotic lesions on leaf veins. *Xoo* can also infect the rice at the seedling stage called kresek and can result in partial or even complete crop loss. Similarly, rice sheath blight caused by soil-borne necrotrophic fungal pathogen *Rhizoctonia solani* (*R. solani)* causes disease in numerous plant species (Ghosh et al., 2021). An infection by *R. solani* causes pre- and post-emergence seedling blight, panicle infection, and spotted seed in rice (Kumar and Amaresh Gouda, 2018).

Numerous resistance genes are used by breeders to develop resistant varieties to control bacterial and sheath blight. Several rice introgression lines using gene pyramiding have been developed, and varieties, such as Samba Mahsuri, BPT5204, Pusa Basmati, have been extensively grown (Joseph et al., 2004; Sundaram et al., 2008). Apart from these strategies, the chemical genomic approaches have also shown the potential to control bacterial diseases (Hicks and Raikhel, 2012). Melatonin, a natural compound, inhibits bacterial cell proliferation by altering the structure of the cell (Acuña-Castroviejo et al., 2001; Chen et al., 2018). Natural phenolic compounds, such as salicylic acid (SA), play a major role in defense against *Xoo* (Fan et al., 2017). Thiazole derivatives inhibit bacterial infection on rice by targeting the type 3 secretory system (T3SS) without altering the bacterial growth (Jiang et al., 2019; Tao et al., 2019). Niclosamide effectively blocks the growth of *Xoo* without hampering the growth of other beneficial bacteria (Sahu et al., 2018). Fungicides, such as Hexaconazole, nano-hexaconazole, Validamycin, and propiconazole, and formulation of Contaf 5 EC was effective against sheath blight caused by *R. solani* (Gopal et al., 2012; Uppala and Zhou, 2018). However, rice cultivation has spurred the emergence of new and more virulent races of *Xoo* or *R. solani* making the chemical means of disease management largely ineffective (Angeles-Shim et al., 2020). Therefore, instantaneous crop protection is a prerequisite to achieve the real yields of the crop.

Cholic acid (CA) is a natural bile acid that is abundantly found in the soil samples due to the fecal matter of animals and is known to have potential antimicrobial activity (Koga et al., 2006). CA plays a significant role in defense response by triggering phytoalexin, induction of pathogenesis-related proteins, and PAMP-triggered immunity (PTI) that confers broad-spectrum resistance against phytopathogens (Koga et al., 2006). CA showed systemic resistance against fungus *Magnaporthe grisea* in rice (Koga et al., 2006), and deoxycholate elicits defense response in *Arabidopsi*s by reducing the growth of *Pseudomonas syringae* pv. *tomato* and *Erwinia amylovora* (Zarattini et al., 2017). As CA is a naturally occurring biomolecule of the human body, CA-derived biodegradable molecules can provide a suitable alternative to mitigate plant pathogens. Our earlier studies have shown that cholic acid-glycine conjugates (CAGCs) are potent broad-spectrum antimicrobials over the naturally occurring bile acids (Yadav et al., 2019, Gupta et al., 2019).

In this study, we present the screening of CAGCs for antimicrobial activity against *Xoo and R. solani* and identified effective CAGC that can completely inhibit the growth of these pathogens. Exogenous application of the most effective C6 CAGC on the infected rice could rescue the rice from disease progression, and also enhanced the plant immunity-associated genes *in planta*. Rice seeds primed with C6 could induce defense genes, providing resistance against *Xoo*. Therefore, the application of CAGCs can be an effective strategy for protecting the crop from leaf and sheath blight diseases on rice.

## Materials and Methods

### Bacterial Strain and Culture Conditions

The *Xoo* strain PXO99^A^ was grown in nutrient broth (NB) medium in a shaking incubator at 28°C. Nutrient broth medium was prepared using peptone (5 g), NaCl (5 g), beef extract (1.5 g), and yeast extract powder (1.5 g) in 1,000 ml of distilled water (pH 7.0–7.2). Nutrient agar (NA) medium was prepared with peptone (5 g), NaCl (5 g), beef extract (1.5 g), yeast extract powder (1.5 g), and agar powder (15 g) in 1,000 ml of distilled water.

### Antibacterial Activities of CAGCs

We synthesized nine different CAGCs as reported previously (Yadav et al., 2019). A single colony of *Xoo* (PXO99) was cultured in NB medium at 28°C with shaking at 180 rpm. The optical density (OD_600_) was measured, and the final OD was set as 0.1. Bacterial culture was then inoculated into a 96-well plate containing 100 μl of compounds that were diluted from higher concentrations (256, 128, 64, 32, 16, 4, 2, 1, and 0.5 μg). Media (200 μl) without bacterial culture and bacterial suspension (200 μl) without CAGC treatment were used as control. The plates were incubated at 28°C, and OD_600_ was measured at an interval of 6 h using a spectrophotometer (MiniMax 300 Imaging Cytometer, Molecular Devices, CA, USA). To determine the lowest antibacterial drug concentration to inhibit 99% bacterial growth, minimum inhibitory concentrations (MIC99) from four biological replicates for each concentrations were measured.

### *In vitro* Antibacterial Assay

The *Xoo* cells (~10^6^ CFU/ml) were treated with 16 μg of C4, C5, and C6 CAGCs and incubated for 0, 1, 2, 4, 8, and 24 h and plated on NB agar medium, and colonies were counted after 48 h of incubation at 28°C. For measuring the bacterial membrane disruptions, the *Xoo* cells were grown in NB medium till logarithmic phase followed by re-suspension in 1X PBS. To the *Xoo* culture, *N*-phenylnapthyl amine (NPN) (Sigma-Aldrich, Cat no. 104043, MO, USA) (2.1 μM) was added, and fluorescence at λ_ex_ of 350 nm and λ_em_ of 420 nm was measured for 30 min using fluorescence spectrophotometer. Once stabilized, the cultures were treated with C4, C5, and C6 CAGCs, and a change in fluorescence was recorded.

For inner membrane permeabilization assay, 3, 3′-diethylthiadicarbocyanine iodide (DiSC_2_) (10 μM) (Sigma-Aldrich, Cat no. 173754) was added to the bacterial suspension (1X PBS), and the reaction was stabilized with 1 mM KCl. Fluorescence at λ_ex_ of 637 nm and λ_em_ of 670 nm was measured using a spectrophotometer. Cultures were then treated with C4, C5, and C6 CAGCs, and a change in fluorescence was recorded.

For propidium iodide (PI) assay, the bacterial suspension of 5 × 10^8^ CFU/ml was treated with CAGC C4, C5, and C6 for 60 min. The CAGC-treated bacterial suspension was then incubated with PI (10 μg/ml) (Sigma-Aldrich, Cat no. P4170) for 20 min at 28°C. The unbound PI in bacterial cells was removed by washing with 1X PBS. The PI stained cells were detected using a flow cytometer (BD Facsverse) and data is represented as a percentage of PI positive cells.

### Colony Forming Units (CFU) Assay

The *Xoo* culture was grown in nutrient broth till log phase (OD = 1) and was used to inoculate biofilms on coverslips. For biofilm growth, sterilized glass coverslips (Bluestar, 18 mm) were soaked in a Petri dish having NB and Tryptic Soy Broth (TSB, 10 ml total volume) in a 1:1 v/v ratio. Inoculation was done from fresh culture (100 μl) and Petri dishes were kept at 37°C for an initial 48 h and then, at 28°C for 5 days. On day 7, the biofilms were washed and treated with C6 CAGC and Kanamycin at 16 and 32 μg/ml separately and kept for 5 h at 37°C. After 5 h, biofilms were washed and trypsinized (1X Trypsin, 100 μl) and suspensions were plated on nutrient agar plates kept at 28°C for 3 days for colonies to appear and expressed as Log_10_ CFU/ml (*n* = 3).

### Viability Staining Assay

Untreated and C6 treated *Xoo* biofilms were developed as described above. After treatment, the washed biofilms were stained with SYTO-9 and PI (mixed in 1:1 v/v) for 20 min and washed with 1X PBS. Then, the biofilms were fixed using 4% paraformaldehyde for 15 min. Further, the biofilms were washed and kept on slides for imaging under a confocal microscope (Leica TCS SP5, Germany). Fluorescein isothiocynate (FITC) and Tertamethylrhodamine (TRITC) channels were used for SYTO-9 and PI, respectively, and images were captured using a 40X oil objective.

### To Quantify the Effect on Exopolysaccharide (EPS) and Xanthomonadin Pigment

The bacterial suspension (10^8^ CFU/ml) was incubated with C4, C5, and C6 CAGCs for 72 h at 28°C at 50 rpm. Cells were removed by centrifugation at 5,000 rpm for 30 min. The supernatant was then dissolved by adding 3 volumes of alcohol followed by incubation at 20°C and the precipitate was pelleted by centrifugation at 5,000 rpm for 10 min. The pellet was dried and weighed. Bacterial culture without any compound was used as a control. Data are represented as EPS inhibition (%) over control (Sahu et al., 2018).

For xanthomonadin, cells were collected by centrifugation and mixed in 1 ml of 100% methanol. Tubes were incubated on a rotary shaker for 10 min in dark. The supernatant was collected after centrifugation at 6,000 rpm for 15 min. Methanol was used as a blank. The xanthomonadin pigment was estimated by taking absorbance at 445 nm (Sahu et al., 2018).

### *R. solani* Sclerotial Growth Prevention Assay

Sclerotial growth prevention assay was performed as previously reported (Swain et al., 2017). Briefly, fresh equal-sized sclerotia (*n* = 5) of *R. solani* strain BRS1 were treated with 1 ml of different concentrations (0.1, 0.5, and 1 mg/ml) of C4, C5, and C6 for 4 h at 28°C. After incubation, the sclerotia were washed thoroughly with sterile water, placed on the fresh potato dextrose agar (PDA) plates and incubated at 28°C to grow. The *R. solani* sclerotia treated with distilled water were used as a control. The fungal growth was measured in terms of diameter (in centimeter) of the fungal colony, at 3 DPI. The experiment was conducted using five sclerotia for each treatment.

### 3-(4, 5-dimethylthiazol-2-yl)-2,5-diphenyltetrazolium Bromide (MTT) Assay for *R. solani*

The pre-grown *R. solani* mycelia were treated either with 1 mg/ml solutions of C4, C5, C6, or with water as control and were incubated for 12 h at 28°C. Subsequently, mycelia were harvested and washed thoroughly with phosphate buffer saline (PBS) buffer (10 mM, pH 7.4). The MTT assay was performed as described previously (Meshulam et al., 1995). Briefly, the mycelia were incubated in 900 μl of PBS buffer and 100 μl of MTT solution (5 mg/ml suspended in PBS buffer) for 90 min in dark. The excess MTT dye was removed by washing and dark formazan as an indicator was extracted by incubating in absolute ethanol at room temperature overnight. Upon centrifugation, the OD_570_ of the supernatant was measured using a spectrophotometer (Biorad Smart Spec-3000, CA, USA).

### Toxicity Studies

The rice seeds of a variety of *Taichung Native 1* (TN1) were soaked overnight with two different concentrations (16 and 150 μg) of CAGCs C4, C5, and C6. In this study, 10 seeds in each concentration were germinated on wet filter paper. Shoot and root length were measured for 5 days from germination. The seeds grown in distilled water were used as a control. For excised leaf disc assay, leaf disc of rice, 1 × 1 cm were incubated overnight with CAGCs C4, C5, and C6. The reactive oxygen species (ROS) levels, lipid peroxidation, and membrane damage were quantified.

### Pathogen Infection Assay on Rice

Rice variety TN1 plants were grown in a growth chamber for 45 days. The bacterial suspension was prepared in MES buffer (10 mM MES, 10 mM MgCl_2_), and 45-day-old leaves were infected with *Xoo* suspension (10^6^ CFU/ml). After 24 h, the infected plants were sprayed with CAGC C6 (16 μg). The bacterial multiplication was analyzed at 96 h of post spray (hps) by plating on the NA plates. In another set of plants, C6 (16 μg) was sprayed and after 24 h, the plants were infected with *Xoo* by leaf clipping method. The grayish to chlorotic symptoms from the top to the edge were measured to assess the disease and lesion length and monitored after 8 days post infection (dpi) and bacterial multiplication was assessed at 96 h post infection (hpi).

### Testing Efficacy of C4, C5, and C6 on Controlling Sheath Blight Disease in Rice

The CAGCs of 1 mg/ml solution of C4, C5, and C6 chemicals were exogenously sprayed on 60 days old PB-1 rice plants. After 12 h of spray, freshly grown *R. solani* sclerotia were inoculated into a rice sheath (Ghosh et al., 2021). After 1 dpi, CAGCs were again exogenously sprayed, and plants were incubated under the control conditions. The necrotic disease lesions on the rice sheaths were recorded at 6 dpi and the relative vertical sheath colonization (RVSC) index was calculated (Ghosh et al., 2021). The experiment was conducted using three different rice pots/treatment and a minimum of four tillers per pot being infected with *R. solani*.

### Seed Priming With CAGCs

Rice TN1 seeds were soaked in 25 and 50 μg of C6 overnight. CA with 50 μg and water-soaked seeds were used as control. On the next day, vacuum infiltration was done at 25 psi for 5 min followed by rapid drying of the seeds. The seeds were stored until the germination. The seeds were grown and maintained in greenhouse conditions. The 30-days-old plants were infected with 10^6^ CFU/ml of *Xoo*. After 5 and 7 dpi, CFU, membrane damage, and MDA quantification was done. After 15 days of infection, disease symptoms were assessed and then, the lesion lengths were measured at 30 dpi. For the *R. solani*, freshly grown sclerotia were inoculated to 3-day-old seedlings. The lesion length was measured after 3 and 5 dpi.

### Diaminobenzidine (DAB) Staining

The leaf tissues were kept overnight in DAB (SRL- Sisco Research Laboratories, New Delhi, Cat no. 17076) solution of 0.1% (w/v) in 200 mM Na_2_HPO_4_ and Tween 20 (0.05% V/V). The tissues were destained with ethanol: glacial acetic acid: glycerol (3:1:1) by boiling at 100°C for 20 min. The tissues were washed in a fresh destaining solution, and images were taken under a microscope. Accumulation of formazan was quantified by measuring absorbance at 450 nm (Kumar et al., 2014).

### Quantification of Malondialdehyde (MDA)

The seedlings and leaf tissues were homogenized in 5% (W/V) trichloroacetic acid (Merck, Cat no. 76-03-9, Germany) followed by centrifugation for 15 min at RT. The supernatant was mixed with an equal amount of 0.5% thiobarbutiric acid (HiMedia, Mumbai, Cat no. RM1594) prepared in 20% (w/v) TCA. The mixture was boiled for 25 min at 100°C and cooled at RT. The supernatant was collected by centrifugation at 7,500 rpm for 10 min and absorbance measured at 532 nm and corrected for non-specific turbidity by subtracting the absorbance_600_. Standard was prepared using 1, 1, 3, 3-tetramethoxypropane (HiMedia, Cat no. RM3776) with different concentrations (Zhang and Huang, 2013).

### Evan's Blue Staining Assay

Leaf tissues were stained in Evan's blue (Sigma-Aldrich, Cat no. E2129) solution in 0.1 M CaCl_2_ (pH 5.6) overnight. The tissues were washed to remove unbound dye. Images were taken under a microscope. For quantification, the tissues were ground in 1% SDS and centrifuged for 5 min at RT, and the supernatant was measured at 600 nm OD (Vijayaraghavareddy et al., 2017).

### Quantitative Real-time PCR (qRT-PCR)

For RNA isolation from plants, total RNA was isolated from frozen samples in liquid nitrogen using TRIzol reagent (Sigma-Aldrich, Cat no. T9424). A total of 2 μg RNA was converted to cDNA using Takara kit (Cat no. 6110A, Takara Bio, CA, USA). The plant defense gene-specific primers were designed to the sequences downloaded from the rice genome annotation project (Supplementary Table S1). The rice actin is used as an internal control for normalization. The transcript levels were measured by using SYBR green (Takara Bio, Cat no. RR820A) in a qRT-PCR machine (ABI-Quant studio 6 Real Time PCR system, ThermoFisher Scientific, Singapore). The expression data was collected and further processed to calculate the 2^−Δ*ΔCT*^ method (Livak and Schmittgen, 2001).

### Statistics

All graphs were prepared in Graph Pad Prism 7 (San Diego, CA, USA). All the experiments were repeated a minimum of three times with similar results. Statistical significance between the samples was calculated using one-way ANOVA (Tukey's multiple comparisons test) or two-way ANOVA (Tukey's multiple comparisons test).

## Results

### CAGCs Are Broad-Spectrum Antimicrobials

**Figure 1:**
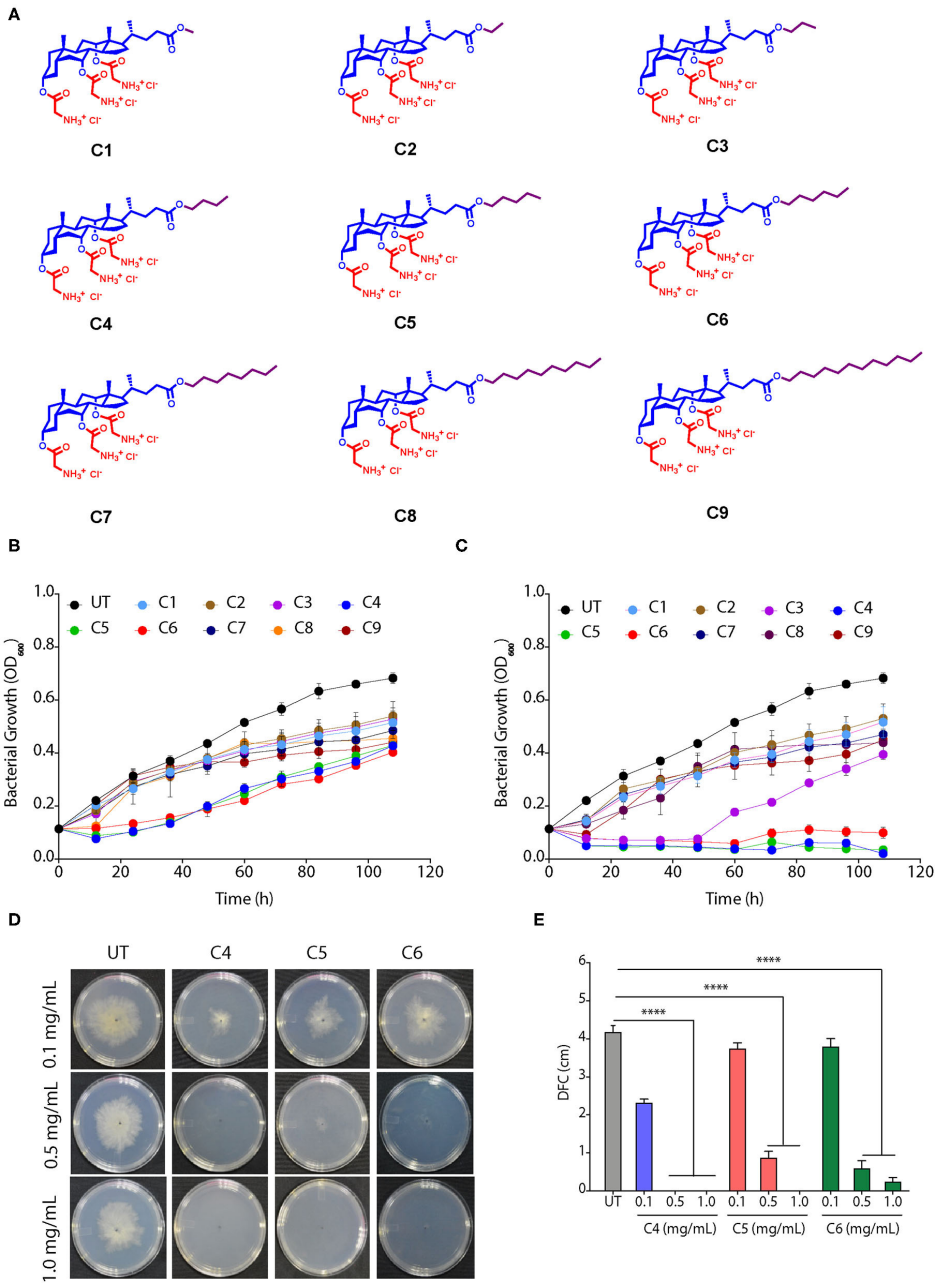
Cholic acid-glycine conjugates (CAGCs) are broad spectrum antimicrobials. **(A)** Molecular structures of CAGCs tested in this study. **(B, C)** Change in growth kinetics of *Xanthomonas Oryzae* pv. *oryzae* (*Xoo)* in presence of **(B)** 8, and **(C)** 16 μg/ml of CAGCs. UT-untreated bacterial culture. The 10^6^ CFU/ml *Xoo* culture was incubated with the CAGCs for different time points and plated on nutrient broth (NB) medium and colony forming units (CFU) were measured. **(D)** Change in sclerotial germination and growth of *Rhizoctonia solani* at 3 days post infection (dpi) after treatment with different concentrations of C4, C5, and C6 CAGCs on potato dextrose agar (PDA) plate. **(E)** Change in diameter of the fungal colony (DFC in cm) during growth of *R. solani* mycelia after treatment with different concentrations of C4, C5, and C6. Graphs show mean values ± SE. A statistical significant difference between indicated groups at *p* < 0.001 (estimated by one-way ANOVA). ****p < 0.001.

We used nine CAGCs, where CA was modified at the carboxylic acid position with different alkyl groups (methyl to dodecyl) using ester bonds, and three glycine moieties were tethered at three hydroxyl groups (Figure 1A) (Yadav et al., 2019). The antimicrobial activity of these nine CAGCs was tested against *Xoo*, and MIC_99_ of CAGCs that are required to kill 99% of bacteria were measured. CAGC, C1, with methyl chain was not effective up to 16 μg/ml, and *Xoo* growth was inhibited at 32 μg/ml for 48 h (Supplementary Figure S1A). Similarly, C2 CAGC with ethyl and C3 CAGC with propyl group showed *Xoo* growth inhibition at 32 μg/ml for only 48 h (Supplementary Figures S1B, C). C4 CAGC with butyl, C5 with pentyl, and C6 with hexyl chains showed *Xoo* growth inhibition at 8 μg/ml (Figure 1B) and was found to be the most effective against *Xoo* with MIC_99_ of 16 μg/ml (Figure 1C and Supplementary Figures S1D–F). In contrast, CAGCs with longer alkyl chains like C7 with octyl chain, C8 with decyl chain, and C9 with dodecyl chain showed partial suppression of *Xoo* growth at 32 μg/ml (Supplementary Figures S1G–I). The structure activity relationship (SAR) studies of CAGCs with *Xoo* revealed that an increase in alkyl chain length at C24 carboxylic acid position up to hexyl chain enhances the bacterial growth inhibition. The growth kinetics at 8 and 16 μg/ml showed that C4, C5, and C6 CAGCs are the most effective antibacterial against *Xoo* as they reduced the multiplication at 8 μg/ml, and complete growth inhibition at 16 μg/ml (Figures 1B, C). To decipher the broad-spectrum antimicrobial activity of CAGCs, we analyzed the effect of C4, C5, and C6 on the growth of fungus *R. solani* that causes sheath blight on rice. All the three CAGCs efficiently suppressed the sclerotial germination, and radial growth of fungal mycelia *R. solani* on PDA plates (Figures 1D, E).

### CAGCs Are Bactericidal

**Figure 2:**
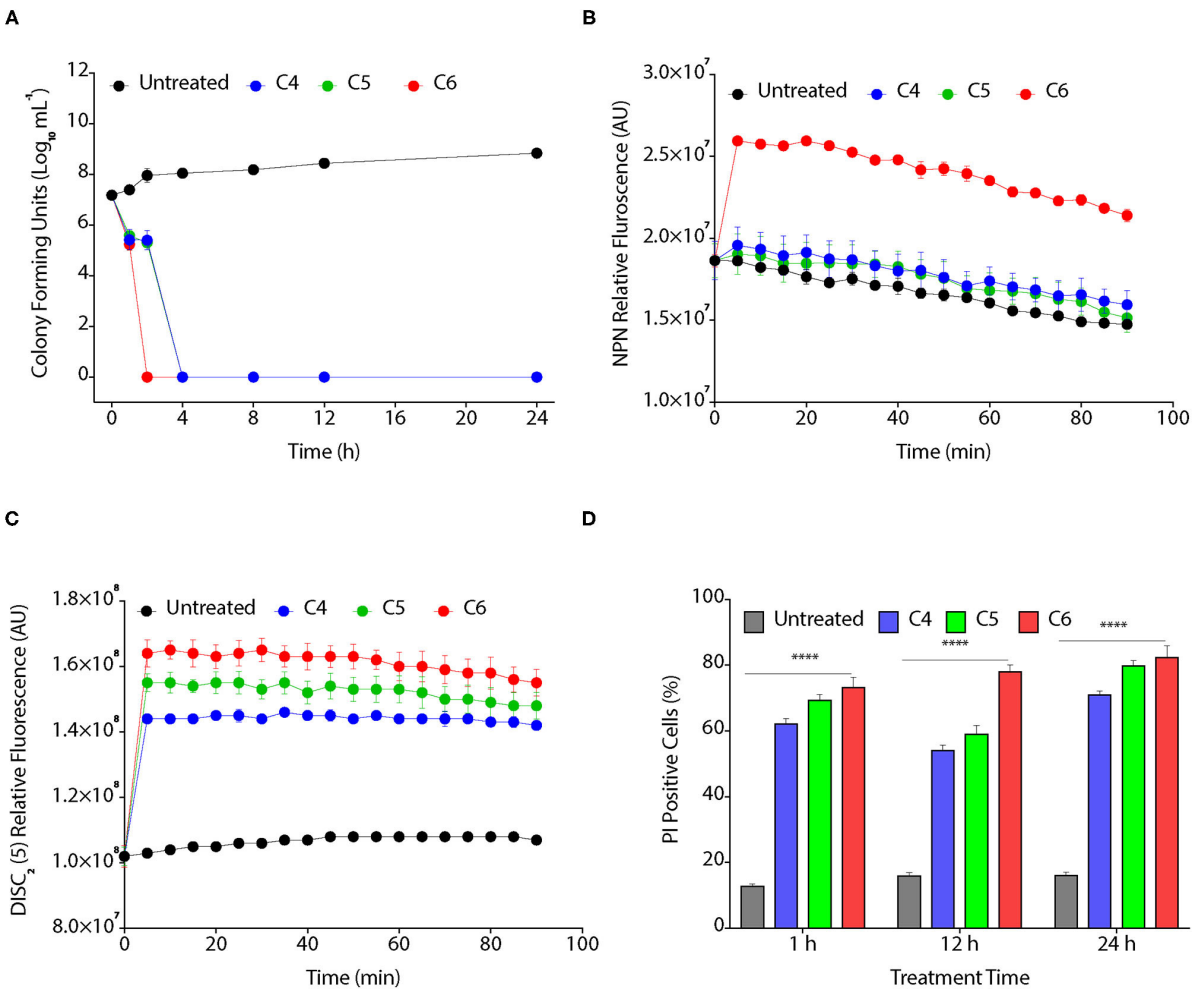
CAGCs affect bacterial membranes. **(A)** Time kill assay on the treatment of *Xoo* with 8 μg/ml of C4, C5, and C6 CAGCs. **(B)** Time-dependent change in fluorescence of *N*-phenyl-1-naphthylamine (NPN) in presence of different CAGCs. **(C)** Change in fluorescence intensity of 3, 3′-diethylthiadicarbocyanine iodide (DiSC_2_) (5) over time after treatment of *Xoo* with different CAGCs. **(D)** Percentage of PI positive *Xoo* cells at a different time on treatment of *Xoo* with 8 μg of different CAGCs. The NPN and DISC_2_(5) fluorescence were measured at λ_ex_ of 350 nm, λ_em_ of 420 nm, and λ_ex_ of 637 nm, λ_em_ of 670 nm by spectrophotometry. The number of *Xoo* live cells with response to CAGCs were measured using FACS. Error bars indicate that experiments were repeated a minimum of three times with four biological replicates. (α = 0.05, ****p < 0.0028). Significant differences were determined by using one-way ANOVA with Tukey's HSD analysis.

We assessed the bactericidal effect of C4, C5, and C6 CAGCs, by time kill assay, as these CAGCs were most effective against *Xoo*. CAGC C6 showed the highest inhibitory effect as no colonies were visible after 1 h of treatment with 16 μg/ml (Figure 2A). In contrast, C4 and C5 CAGCs at 16 μg/ml took 4 h to clear the bacterial growth (Figure 2A). To decipher the bactericidal activity of CAGCs, we assessed the ability of C4, C5, and C6 CAGCs to permeabilize the outer and inner bacterial membranes. The non-polar probe *N-*phenylnapthylamine (NPN) emits weak fluorescence in the aqueous environment, and cell membrane disruptions can allow the NPN to enter the hydrophobic environment that increases its fluorescence. A substantial increase in fluorescence on treatment to *Xoo* with C6 CAGC confirmed its ability to disrupt and permeabilize the outer bacterial membranes (Figure 2B). To estimate the inner bacterial membrane depolarization, we used a membrane potential sensitive cationic dye, [DiSC_2_ (5)] that accumulate in lipid membranes of hyperpolarized cell membranes, and its fluorescence is quenched. Interestingly, the treatment with C4, C5, and C6 CAGCs makes the bacterial membrane depolarized leading to dequenching of accumulated dye and increase its fluorescence (Figure 2C). To assess the membrane disruptions that resulted in the release of intracellular components from bacteria, we estimated the accumulation of membrane impermeable dye, PI, in CAGC-treated *Xoo* cells using flow cytometry. Treatment of C4 and C5 CAGCs at 16 μg/ml showed ~11 and ~18% PI positive cells after 1 h of treatment, whereas C6 at 16 μg/ml showed ~30% PI positive cells (Figure 2D). Therefore, these results suggest that CAGCs are broad-spectrum antimicrobials, and are bactericidal in nature. Similarly, MTT assay showed that C4, C5, and C6 treated *R. solani* mycelia have lost viability, as they were unable to reduce MTT into formazan, a colored compound, whereas the untreated fungal mycelia (water treated) were able to produce colored compound (Supplementary Figure S2A). Quantification of formazan suggested that C4, C5, and C6 CAGCs significantly reduce the viability of *R. solani* mycelia, thereby confirming the fungicidal effect (Supplementary Figure S2B).

### CAGCs Can Degrade Biofilms

**Figure 3:**
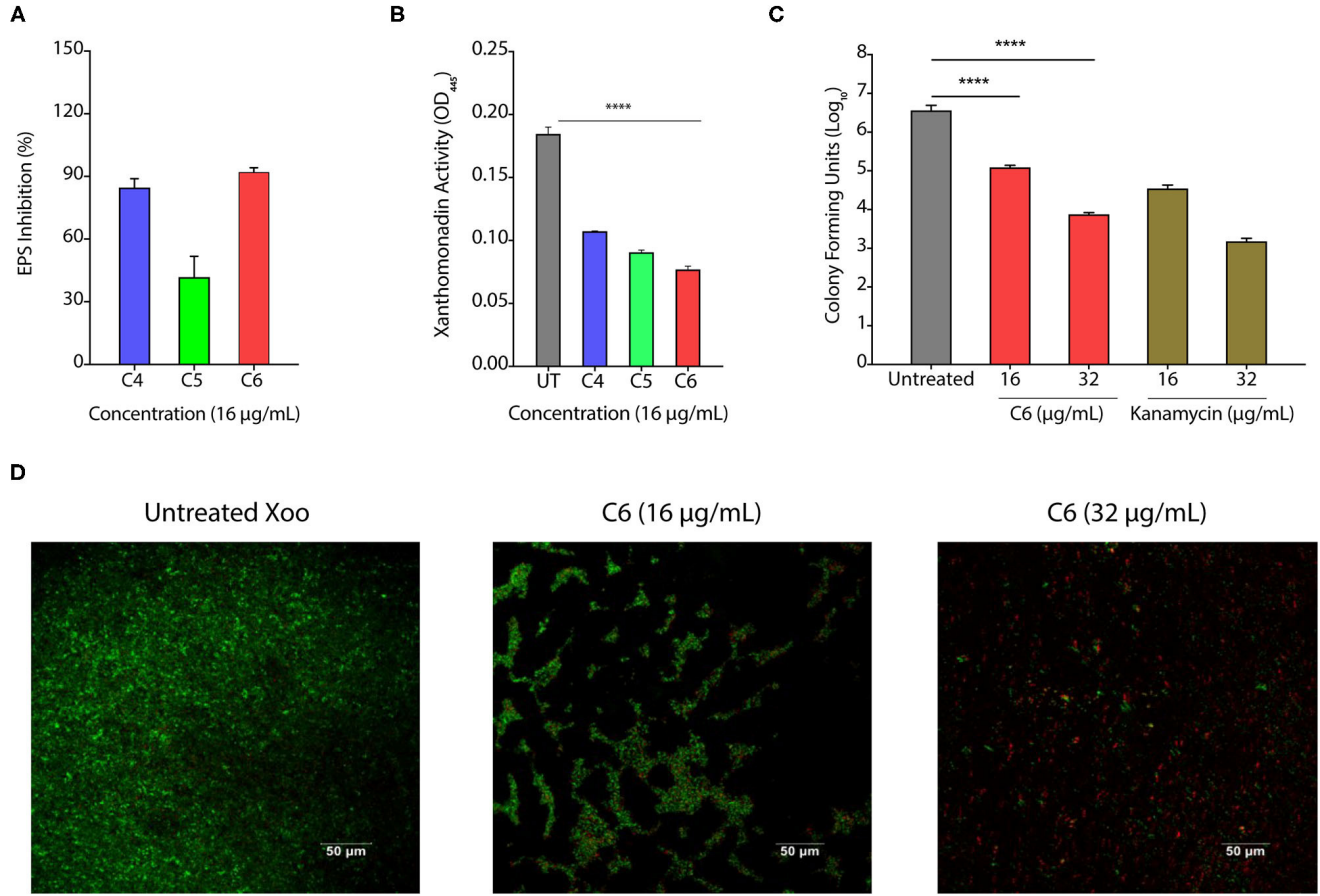
CAGCs can degrade bacterial biofilms. **(A, B)** Percentage change in exopolysaccharide substance (EPS) **(A)** and change in *Xanthomonad* content (α = 0.05, ****p < 0.0001) **(B)** after treatment with **C4**, **C5**, and **C6** CAGCs. **(C)** Change in CFUs of *Xoo* biofilms before and after treatment with **C6** and Kanamycin after 7 days of treatment (**** indicates *p* < 0.0001). Statistical significant difference between indicated groups at *p* < 0.001 (estimated by one-way ANOVA). **(D)** SYTO-PI-stained fluorescence micrographs of untreated and **C6** treated *Xoo* biofilms. Confocal microscopic images were taken after 7-days treatment.

The biofilm is a multilayer matrix-enclosed structure generally tolerant to host defense responses, antibiotics, and environmental stress. Biofilms help in the adherence of bacteria, facilitate colonization, and disease progression in the host (Costerton et al., 1995; Rigano et al., 2007). As CAGCs are bactericidal against *Xoo*, we hypothesized that CAGCs can also degrade the *Xoo* biofilms and inhibit bacterial adhesion on the leaf surface. Biofilms allow the production of xanthan pigment and EPS that protect the bacteria from photochemical damage (Rajagopal et al., 1997; Angeles-Shim et al., 2020). Therefore, we first tested the effect of C4, C5, and C6 CAGCs to inhibit Xanthan and EPS production that are essential for the growth and development of biofilms. C6 CAGC at 16 μg/ml showed the highest inhibition of EPS (Figure 3A) and xanthan production (Figure 3B). We then tested the effect of C6 CAGC on CFU in biofilms and observed a 1-log reduction in bacterial CFU after treatment with 16 μg/ml of C6, and 2-log reduction in CFU at 32 μg/ml of C6 (Figure 3C). SYTO-PI staining of untreated and C6-treated biofilms revealed that untreated *Xoo* biofilms showed a continuous matrix of green fluorescent SYTO-9 stained cells (Figure 3D). The C6 treated biofilms at 16 and 32 μg/ml witnessed a degraded biofilm matrix, and an increased number of PI-stained cells (Figure 3D). A higher number of PI-stained cells (red fluorescence) in the treated biofilms is indicative of killing the biofilm encased bacteria. These results clearly witness the potential of C6 in degrading the pre-formed biofilms of *Xoo* at relatively lower concentrations, and its ability to reduce the xanthan and EPS formation.

### CAGCs Are Non-Toxic to Rice Seedlings and Plants

The pesticides are usually toxic in nature and can negatively affect the growth and development of the plant. Therefore, to study the toxic effect of CAGCs, TN1 seedlings, susceptible for *Xoo*, were soaked with lower (16 μg/ml) and higher concentrations (150 μg/ml) of C4, C5, and C6 CAGCs overnight, and the seeds were allowed to germinate (Supplementary Figures S3A,E). The shoot length of the seeds treated with these CAGCs remains unaffected at 16 μg/ml, the concentration required for combating *Xoo* infection, and gets reduced by 30% at 150 μg/ml (Supplementary Figures S3B,F). Similarly, the root length in CAGC-treated seedlings was unaffected at 16 μg/ml and reduced to 30% only upon treatment with C5 and C6 at 150 μg/ml (Supplementary Figures S3C,G). As pesticide stress can generate ROS and damage the cell membranes, we also studied the effect of CAGCs on cell membrane stability. We estimated the extent of lipid peroxidation end product, malondialdehyde (MDA), accumulated in the CAGC-treated rice seedlings. C4 and C6 treated rice seedlings showed no significant cell membrane damage at both concentrations (Supplementary Figure S3D). However, C5 treated seedlings showed higher levels of MDA accumulation at 150 μg/ml, and the damage was not significant at 16 μg/ml (Supplementary Figure S3H).

We also tested the toxic effect of CAGCs on leaves of 45-days-old grown plants when the plant is prone to infections. The rice leaves were soaked with 16 and 150 μg/ml of C4, C5, and C6 CAGCs overnight, and ROS induction was measured using DAB that stains H_2_O_2_ levels (Supplementary Figures S3I,L). None of the CAGCs showed any remarkable accumulation of H_2_O_2_ in rice leaves (Supplementary Figures S3J,M). We also did not observe any lipid peroxidation at both concentrations (Supplementary Figures S3K,N). Therefore, these results confirm that CAGCs are not toxic to plants.

### CAGCs Protect the Plants From Infections

**Figure 4:**
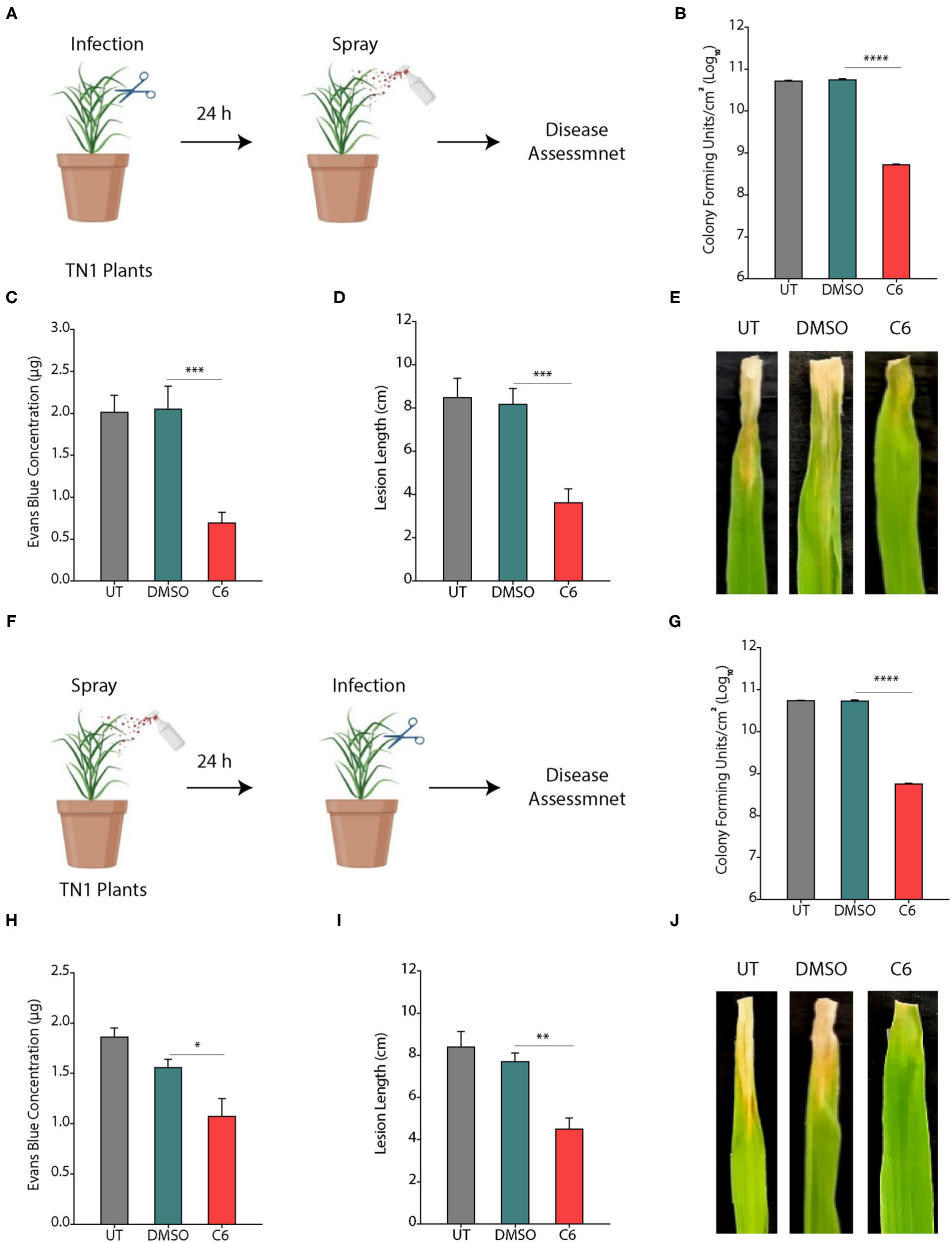
CAGCs protect plants from *Xoo* infections. **(A)** Scheme showing outline of the study where pre-infection with *Xoo* was followed by treatment with C6 CAGC. **(B)** Change in CFUs of *Xoo* on treatment with **C6** (α = 0.05, ****p < 0.0001). **(C)** Membrane stability assessment by Evan's blue dye quantification in untreated and C6-treated *Xoo*-infected rice leaves (α = 0.05, ***p < 0.001). **(D)** Change in pathogen induced lesion length in untreated and C6-treated *Xoo*-infected rice leaves (α = 0.05, ***p < 0.001). **(E)** The photographs showing bacterial leaf blight symptoms on TN1 rice leaves treated with C6. The images were taken after 10 days of *Xoo* infection. **(F)** Scheme showing outline of the study, where treatment was followed by infection with *Xoo*. **(G)** Change in CFUs of *Xoo* on treatment with C6 CAGC (α = 0.05, ****p < 0.0001). **(H)** Membrane stability assessment by Evan's blue dye quantification in untreated and C6-treated *Xoo*-infected rice leaves (α = 0.05, *p < 0.05). (**I**) Change in pathogen induced lesion length in untreated and C6-treated *Xoo*-infected rice leaves (α = 0.05, **p < 0.01). **(J)** Photographs showing bacterial leaf blight symptoms on *Xoo*-infected TN1 rice leaves treated with C6. Graphs show mean values ± SE. A statistically significant difference between indicated groups at *p* < 0.05 (estimated by one-way ANOVA).

To assess the effect of CAGCs on *Xoo* infection on the rice, we selected C6 CAGC as it showed the maximum bactericidal activity with the least toxicity on the rice seedlings and excised leaves. In the pre-infection strategy, 45-day-old TN1 rice leaves were infected with *Xoo* (~1 × 10^6^ CFU/ml). After 24 h of infection, C6 CAGC (16 μg/ml) was sprayed on the infected plants (Figure 4A). CFU analysis showed ~2-log reduction in bacterial load in the C6 treated samples compared with untreated and DMSO treated after 96 hps (Figure 4B). Pathogen-induced H_2_O_2_ and membrane damage in untreated and DMSO treated plants was increased by >4-fold compared with C6-treated leaves as quantified by Evan's blue staining (Figure 4C and Supplementary Figures S4A, B). There was also a 5-fold reduction in the pathogen-induced lesion length on C6 sprayed plants compared with the untreated plants (Figures 4D, E). These results clearly suggest that C6 CAGC spray after infection could effectively inhibit the *Xoo* growth on host plants.

In the post-infection approach, we sprayed C6 CAGC (16 μg/ml) to 45-day-old TN1 rice leaves (minimum five leaves in each group). After 24 hps, leaves were infected with 1 × 10^6^ CFU/ml of *Xoo* (Figure 4F). The bacterial multiplication in C6 sprayed plants after 96 hpi was reduced by >3-log compared with the untreated plants (Figure 4G). H_2_O_2_ levels in C6 treated plants were reduced and membrane stability at 96 hpi in C6 treated plants was increased by >2-fold compared with the untreated plants (Figure 4H and Supplementary Figures S4A, B). Plants sprayed with C6 also showed significantly less disease symptoms with reduced lesion length (Figure 4I) and leaf yellowing symptoms compared with the untreated plants (Figure 4J). The results suggest that C6 spray prior to infection can also inhibit bacterial growth. Comparison of pre- and post-treatment strategy showed the positive correlation in reducing the lesion length (*R*^2^ = 0.9862), pathogen-induced membrane damage (*R*^2^ = 0.8367), and bacterial multiplication (*R*^2^ = 0.9996) (Supplementary Table S2).

Similarly, we also tested the effect of CAGCs on *R. solani* infection in rice plants. Rice plants were sprayed with 1 mg/ml of C4, C5, and C6 CAGCs, and subsequently infected with a pathogen. Compared with the control plants, CAGC treated plants showed higher levels of protection against *R. solani*. Among them, C6 treatment was most effective as the size of necrotic disease lesion was significantly reduced compared with others (Supplementary Figure S4C). The disease index in terms of RVSC was significantly reduced in C6 treated samples (Supplementary Figure S4D). Therefore, these results clearly witness that C6 CAGC can improve crop protection effectively by inhibiting *Xoo and R. solani* infection.

### CAGCs Activates Plant Defense Responses

**Figure 5:**
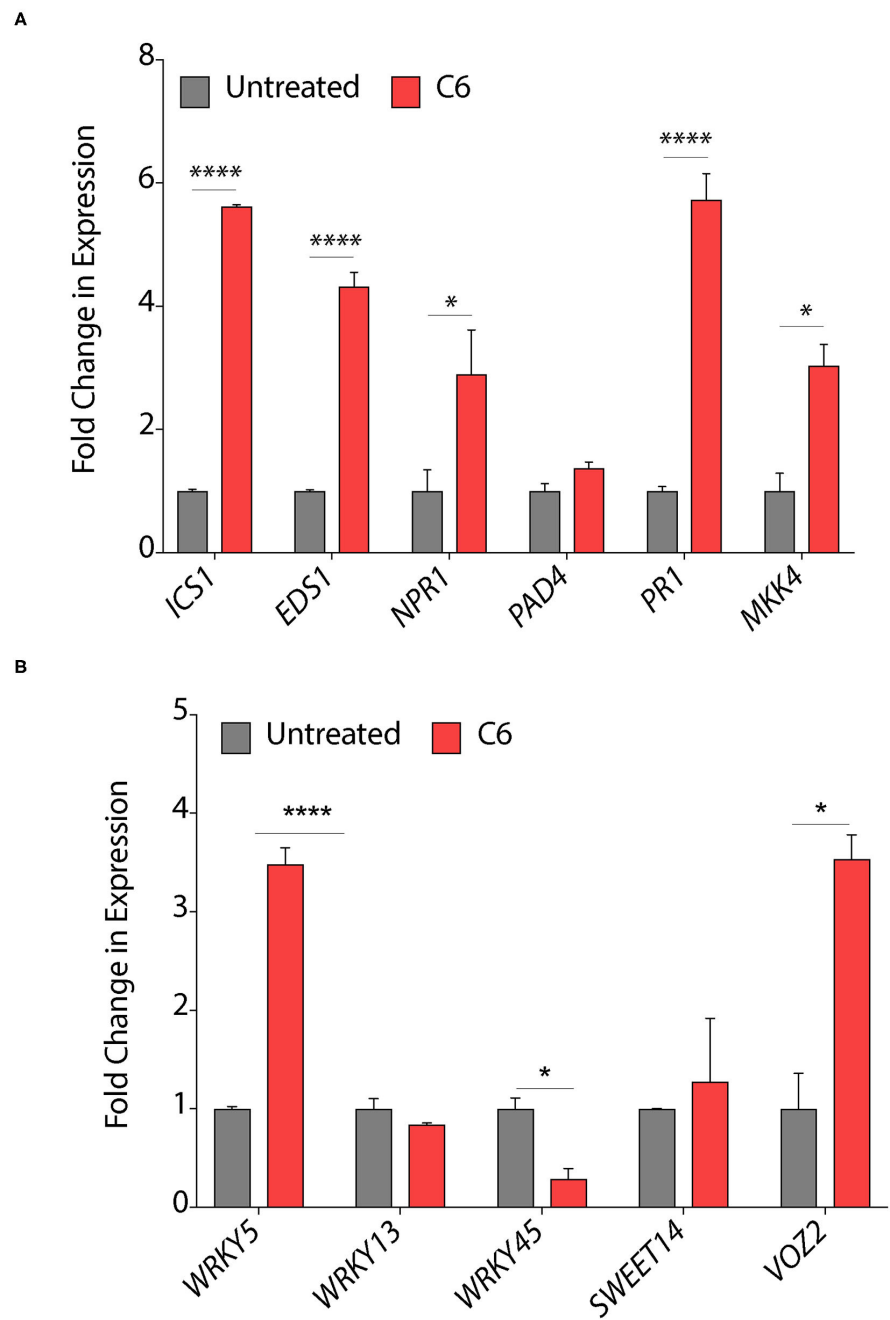
Response of plant defense genes upon CAGCs treatment. **(A, B)** Change in expression of plant immunity associated genes in response to *Xoo* and C6 treatment. The expression of SA signaling and pathogen related genes were performed from leaf samples infected with *Xoo* followed by C6 treatment after 48 hps. Minimum three biological replicates were used for each time point and experiments were repeated three times with similar data. Statistical significance was performed with two-way ANOVA (α = 0.05, *p < 0.05, ****p < 0.0001).

Chemicals, such as SA and jasmonic acid (JA), are known to induce the expression of plant defense responsive signaling genes, pathogenesis-related genes, and can also modulate several transcription factors (Mur et al., 2008). Secondary bile acid and deoxycholic acid showed induction of defense related genes, reprogramming of transcription, callose deposition, ROS production, JA and SA signaling pathways in *Arabidopsis* with response to *Erwinia amylovora* and *Pseudomonas syringae* pv. *Tomato* (Zarattini et al., 2017). Therefore, to decipher the effect of C6 CAGC on gene expression, we quantified the expression of these genes in pre-infection strategy as it mimics the field conditions. Treatment of C6 CAGC induced >5-fold increase in transcripts of SA biosynthetic pathway gene *ICS1* (*isochorismate synthase)*, >4-fold increase in transcripts of *EDS1* (*enhanced disease susceptibility)*, and >3-fold increase in the transcripts of non-pathogenesis related genes *NPR1 (nonexpressor of pathogenesis-related genes)* compared with untreated *Xoo*-infected plants, whereas there were no significant changes in the transcript levels of *PAD4* (*phytoalexin deficient*) (Figure 5A). We also observed a >6-fold increase in the transcript levels of the *PR1* gene and >3-fold increase in expression of Mitogen-activated protein kinase 4 (*MKK4*) after treatment with C6 compared with the untreated *Xoo*-infected plants (Figure 5A). The transcripts of transcription factors that acts as positive regulator of defense, such as *WRKY5* were induced by >3 fold, whereas no significant change was observed in *WRKY13* (Figure 5B). In contrast, *WRKY45* transcript levels were reduced by 2-fold after 48 hps in the C6 treated plants compared with the untreated *Xoo*-infected plants (Figure 5B). Repression of WRKY13 with response to C6 could positively regulate SA-pathway-dependent disease resistance against *Xoo* as similar to *M. oryzae* (Qiu et al., 2007). The expression of *WRKY* TFs at early time points after infection triggers SA related signaling genes *PAD4, NPR1, ICS1, EDS1, and PR1* in the C6 CAGC treatment with response to *Xoo*. *Xoo* secretes TAL effectors which target *SWEET14* involved in sugar transport and transcription factor *VOZ2* to hijack plant immunity (ZhiYuan et al., 2018). The transcripts *of SWEET14* and *VOZ2* were >2-fold increase in the C6 treated plants compared with the untreated plants (Figure 5B). Despite higher *SWEET14* or *VOZ2* transcripts, C6 CAGC could protect the rice plants from *Xoo* infection by SA mediated defense pathway.

### Seed Priming Protects Rice Plants From Infection

**Figure 6:**
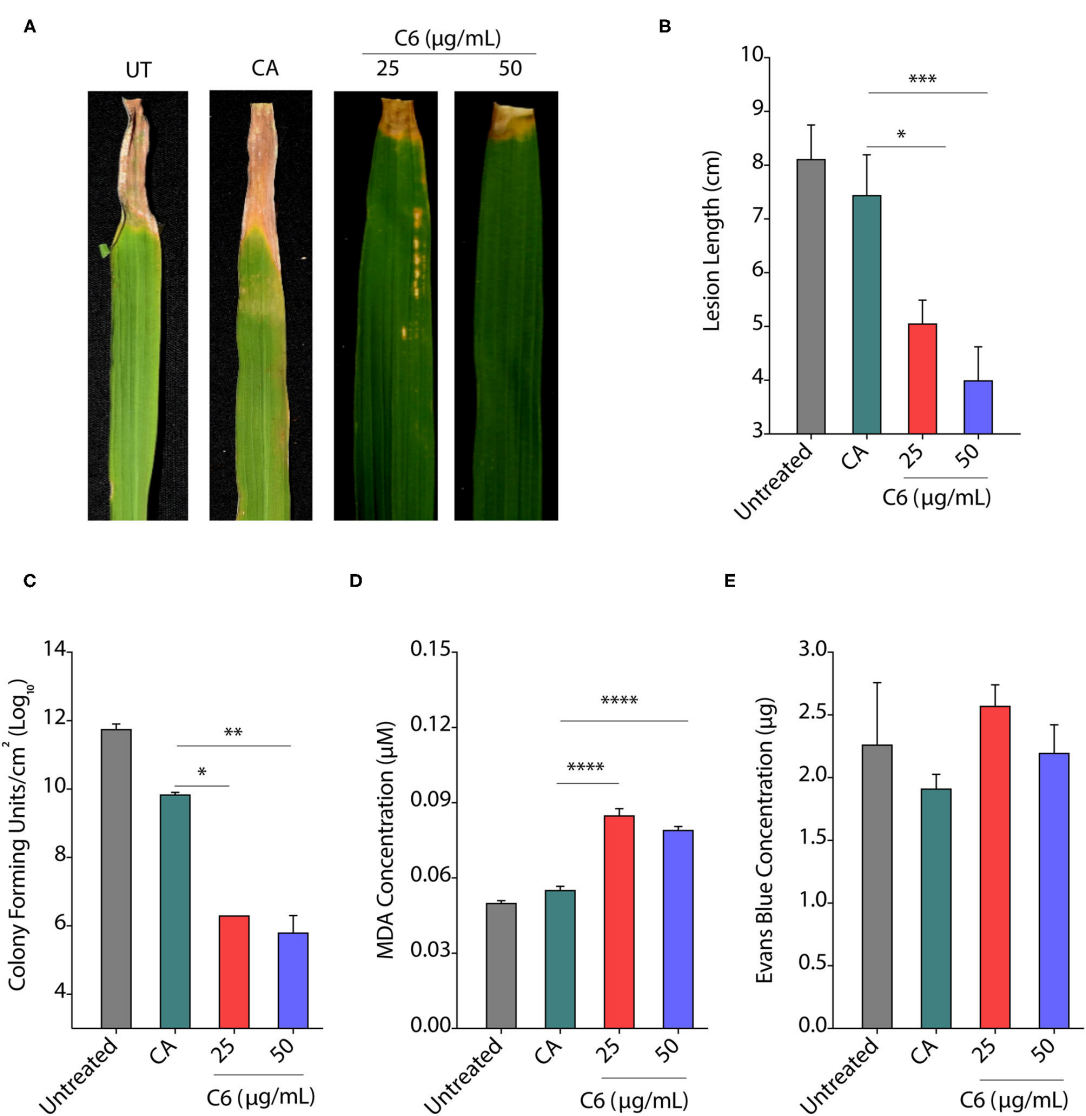
Seed priming provides protection from infection. **(A, B)** Pictures showing the bacterial disease symptoms at 15 dpi **(A)** and change in lesion length at 30 dpi **(B)** after priming of rice seedlings with C6 CAGC. **(C–E)** Change in CFUs **(C)**, lipid peroxidation derived malondialdehyde (MDA) levels **(D**), and membrane stability assessment by Evan's blue dye quantification **(E)** of infected leaves at 5 dpi after priming with C6 at 25 and 50 μg/ml. For priming, rice seeds were soaked in C6 at 25 and 50 μg/ml overnight followed by vacuum infiltration for 5 min, and seedlings were established in the soil. About 30 day-old plants were infected with *Xoo* (10^6^ cfu/ml). The experiment was repeated three times with minimum of 10 biological replicates. Values are means + SE from three biological replicates. A significant difference was determined by using two-way ANOVA with Tukey's HSD test (α = 0.05, * *p* < 0.05, **p < 0.01, ***p < 0.001, ****p < 0.0001).

Priming defense response of plants by seed treatment is one of the attractive practices to protect them from infection. Plant hormones and natural compounds, such as Strobilurins or seaweed-derived stimulants can induce the priming effect (Nair et al., 2012; Filippou et al., 2016; Kerchev et al., 2020). The priming of seeds can lead to improved seed germination, seedling emergence, and productivity in crop species with improved resistance against biotic and abiotic stress (Khan et al., 2020). Therefore, the molecules stimulating plant defense without affecting plant growth are essential for the successful establishment of good crop growth. To decipher the effect of seed priming by CAGCs, TN1 rice seeds were incubated with 25 and 50 μg/ml of C6 CAGC overnight followed by vacuum infiltration and drying seeds rapidly. The priming of seeds with C6 CAGC did not exhibit any unexpected growth defect (Supplementary Figure S5). Seedlings were grown in soil for 30 days before infection with *Xoo* (10^6^ CFU/ml). We observed that disease symptoms on leaves were significantly reduced in C6 primed plants after 15 dpi compared with water and only CA-primed plants (Figure 6A). We observed a >2-fold reduction in lesion length in C6-primed plants even after 30 dpi (Figure 6B). Quantification of CFUs showed a >4-log reduction in bacterial growth after 5 dpi in C6 seed primed leaves compared with unprimed leaves (Figure 6C). We observed a 2-fold increase in lipid peroxidation end product MDA at 5 dpi in the seed primed rice plants after pathogen infection (Figure 6D). These reactive carbonyl species may kill the pathogen effectively. Despite higher MDA levels, membrane stability as quantified by Evan's blue dye after pathogen infection in seed-primed plants was not significantly affected as compared with the unprimed or CA treated plants (Figure 6E). Similarly, we observed that CAGC-primed seedlings also exhibited resistance against *R. Solani* after 3 dpi with less lesion length (Supplementary Figures S4E, F).

### Priming With C6 Enhances Defense Associated Gene Expression in Rice

**Figure 7:**
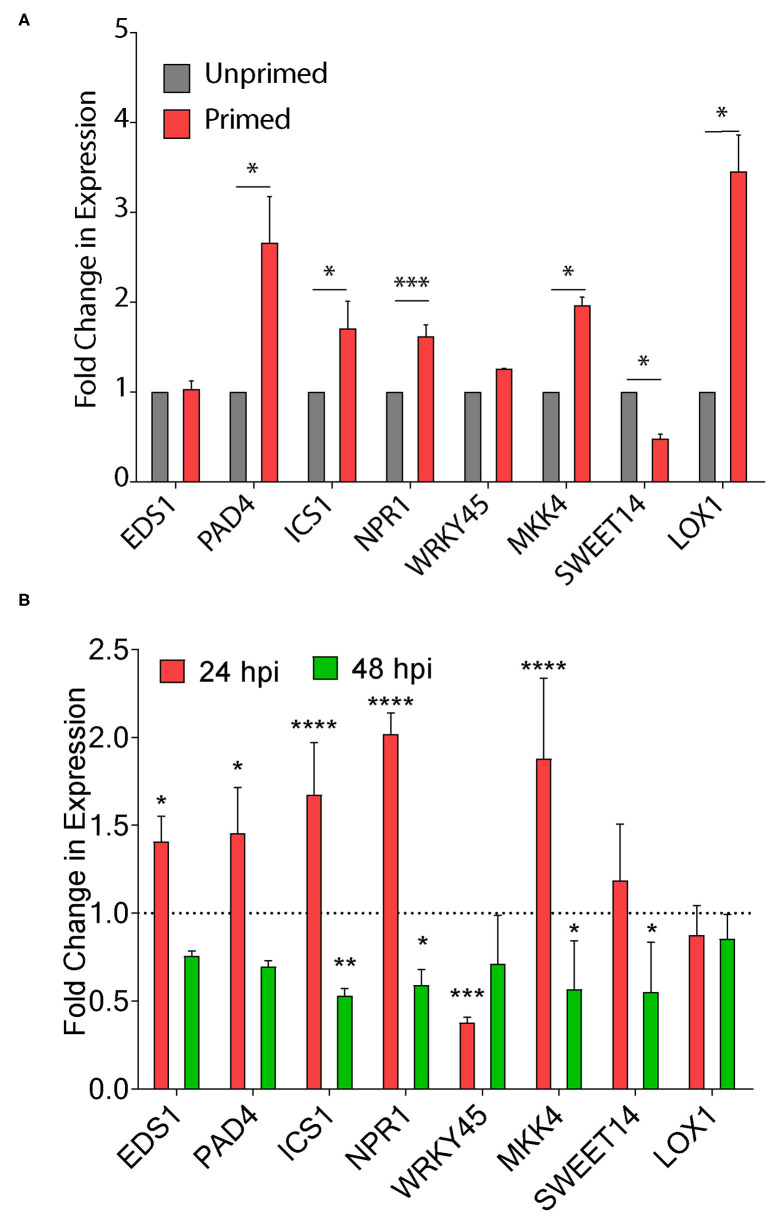
CAGC induces plant defense responsive genes. **(A, B)** Change in expression of plant immunity associated genes upon seed priming **(A)** and *Xoo* infection in seed primed plants **(B)**. The TN1 rice seedlings were primed with C6 (50 μg) and water-soaked seeds were used as control. The 30 days-old seed-primed plants were infected with *Xoo* (10^6^ cfu/ml). The leaf samples were collected 24 h before the infection and after 24 and 48 hpi and total RNA was isolated. The cDNA was prepared and used for qRT-PCR analysis. Values are means + SD from three biological replicates. A significant difference was determined by using two-way ANOVA with Tukey's HSD test (α = 0.05, * *p* < 0.05, **p < 0.01, ***p < 0.001, **** *p* < 0.0001).

Seed priming is known to improve the defense response in plants by triggering the genes involved in disease resistance (Kerchev et al., 2020). The chemical inducers benzothiadiazole and probenazole trigger plant fitness through the activation of the SA pathway inducing resistance *via* the priming effect (Takatsuji, 2014). Therefore, we first quantified the change in expression of defense response genes on seed priming after 30 days. We did not observe any change in the expression of *EDS1* in C6 primed plants, whereas there was a >2-fold increase in transcripts of SA dependent *PAD4* and *ICS1* genes upon seed priming (Figure 7A). Similarly, we observed a two-fold increase in nonpathogenesis related genes *NPR1* transcripts upon seed priming. Transcription factor *WRKY45* transcript levels remained unchanged, and there was a >2-fold increase in MKK4 expression (Figure 7A). We also observed a three-fold increase in expression of the *LOX1* gene involved in JA biosynthesis upon priming (Figure 7A). Surprisingly, we observed a >4-fold decrease in expression of *SWEET14* on seed priming.

Next, we infected the 30-day-old TN1 seed unprimed and primed plants and quantified the changes in expression of these genes in *Xoo* infected plants at 24 and 48 hpi. There is a >2-fold increase in *EDS1* transcripts after 24 hpi with no change at 48 hpi (Figure 7B). We did not observe any significant change in SA dependent *PAD4* and *ICS1* genes at 24 and 48 hpi (Figure 7B). The role of *NPR1 and PR1* in priming the defense response in rice and Arabidopsis was reported (Takatsuji, 2014). *NPR1* transcripts were increased by >2-fold at 24 hpi, and reduced at 48 hpi, whereas *WRKY45* transcripts were reduced by 4-fold at 24 hpi with no change at 48 hpi (Figure 7B). Expressions of MKK4, *SWEET14*, and *LOX1* remain unchanged. These results clearly demonstrate that seed priming of CAGCs could trigger the plant immunity associated genes. CAGCs can modulate the transcriptional signaling involved in the enhanced defense mechanism to protect plants from diverse pathogens.

## Discussion

Bile acids are bioactive molecules, and the presence of a hydrophilic side chain and carboxylic group on the hydrophobic backbone favors the tethering of required ligands to fine tune their activity. Bile acid derivatives can be synthesized easily and can act as effective antimicrobial compounds (Lai et al., 2008). CA (a kind of bile acid) is abundantly found in soil samples as a part of fecal matter generated from animal sources (Mendelski et al., 2019). CA-derived conjugates have been studied extensively for their antimicrobial activity against different Gram-negative bacteria, such as *Escherichia coli* (*E. coli*), *Pseudomonas aeruginosa, Klebsiella pneumonia*, and *Acinetobacter baumanii, and* Gram-positive bacteria (Kumar et al., 2019; Mitra et al., 2019), and *Candida albicans* fungal models (Gupta et al., 2019). However, CA or CAGCs have not been exploited in agriculture even though they are found to be abundant in soil. Our studies showed that C6 CAGC with hexyl chain is bactericidal against *Xoo* and fungicidal against *R. solani* by disrupting its membranes as reported in other microbial systems (Yadav et al., 2019). CAGCs degrade and reduce >60% biofilm formation at lower concentrations that inhibit the ability of *Xoo* to secrete *Xanthomonadin* required for the attachment to the leaf surface, *Xoo* viability, and pathogenesis (Park et al., 2009; He et al., 2011; Yu et al., 2015). Antibacterial compounds, such as melatonin and niclosamide are known to inhibit the biofilms and can effectively suppress bacterial survival on the host. Compounds, such as niclosamide (Kim et al., 2016; Sahu et al., 2018) and 1, 3, 4-oxadiazole are also reported to regulate the EPS production in *Xoo* (Shi et al., 2015). The CAGCs could effectively inhibit the biofilms by reducing EPS and Xanthan.

The hypersensitivity response caused by *Xoo* on *N. benthamiana* was inhibited with the treatment of CAGC that could kill the pathogen, thereby suggesting that CAGC induced ROS could counterattack the pathogen. ROS accumulation can trigger the expression of defense genes in protecting the rice plants from *Xoo or R. solani* infection, and this elicitation of ROS due to CAGCs may act as a defense against diverse pathogens (Liu et al., 2010; Ali et al., 2018). The exogenous application of C6 CAGC against *Xoo* or *R. solani* either pre- and post-infection spray on rice showed effective inhibition of bacterial and fungal growth and reduced lesion length on rice leaves and stem. The reduced pathogens multiplication and lesions on leaves and stems suggest that CAGC molecules could protect the rice plants as equivalent to genetically enhanced plants introgressed with *Xa* alleles or gene-edited plants (Oliva et al., 2019). *R. solani* is a catastrophic fungal pathogen because of its complex lifestyle and coenocytic nature, and developing resistance against this pathogen is a challenging task (Srinivasachary and Savary, 2011). The CAGCs tested against *R. solani* could effectively prevent the sclerotia growth and kill the pathogen as evidenced by the MTT assay. The exogenous application of CAGCs could effectively reduce the infection caused by *R. solani*, and ROS induced due to the CAGC treatment may suppress the *R. solani* colonization as we observed from our earlier studies (Kant et al., 2019).

Secondary bile acid, deoxycholic acid showed induction of defense related genes, reprogramming of transcription, callose deposition, ROS production, JA and SA signaling pathways, induction of antimicrobial compounds, and phytocassanes in Arabidopsis with response to *Erwinia amylovora* and *Pseudomonas syringae* pv. *tomato* (Zarattini et al., 2017). The C6 induced defense gene expression in rice further suggests that ROS could trigger the upregulation of many genes involved in defense response against *Xoo* infection. The transcription factor WRKY13 repression in response to C6 could positively regulate SA-pathway-dependent disease resistance against *Xoo* as similar to *M. oryzae* (Qiu et al., 2007). Overexpression of *indica WRKY45* (*WRKY45-2*) showed resistance, whereas *japonica*- *WRKY45* (*WRKY45-1*) showed susceptibility to *Xoo* (Tao et al., 2009). The expression of WRKY TFs at early time points after infection directly influenced the induction of SA related signaling genes *PAD4, NPR1, ICS1, EDS1, and PR1* in the C6 CAGC treatment with response to *Xoo*. *NPR1* is involved in priming the defense response in rice and Arabidopsis (Takatsuji, 2014). Induction of *PR1* with response to CA against *Magnaporthe grisae* was reported (Koga et al., 2006). The chemical inducers, such as benzothiadiazole and probenazole, trigger plant fitness through the activation of the SA pathway inducing resistance *via* the priming effect (Takatsuji, 2014). This study demonstrates that C6 could elicit plant defense through SA mediated pathway. The *Xoo* effector targets *SWEET14 a* sugar transporter was induced with response to *Xoo* in C6 treatment that was known to hijack plant immunity (ZhiYuan et al., 2018). In spite of induction of *SWEET14* or *VOZ2* transcripts, C6 could be able to protect the rice plants from the *Xoo* infection by SA mediated defense pathway or by directly disrupting the bacterial membrane. The CAGC derived molecules can be degraded by certain bacteria easily (Philipp, 2011) and can be adopted in agriculture for crop protection.

When the rice seeds are primed with a CAGC molecule, they could induce the expression of many defense responsive genes before pathogen infection. The higher transcripts level of SA related genes like *ICS1, EDS1*, and *PAD4* were upregulated before infection with *Xoo*. Even after pathogen infection after 24 h, the genes were induced at higher levels except *WRKY45* suggesting that the priming effect with CAGC could maintain the host defense strategy against pathogens. The early trigger of these pathogen defense genes can have improved memory to activate the defense process in plants and for subsequent time hours, those genes are not required for defense. *SWEET14* transcripts were suppressed before pathogen infection and the pathogen could not hijack host machinery for its multiplication. The induction of *SWEET14* was not observed even after pathogen infection could be due to the disruption activity of CAGCs on the bacterial membranes, hence no effect of bacterial effector triggering the plant genes to cause virulence. The CAGCs could also stimulate the expression of JA biosynthesis gene *LOX1*. Higher expression of *LOX1* is associated with crop protection against necrotropic and oomycete pathogens, such as *R. solani* (Kumar et al., 2013).

Overall, we screened nine CAGCs against *Xoo* and identified three CAGCs showing higher antimicrobial effects. Characterization of these CAGCs revealed that C6 with hexyl chains could effectively disrupt the bacterial membranes and biofilms. These CAGCs have less or no toxic effect on rice seedlings. Foliar application of C6 CAGC could effectively protect rice from *Xoo or R. solani* infection. The SA dependent defense genes were triggered in C6 CAGC treated plants. Rice seeds primed with C6 CAGC showed improved resistance to pathogen infection even after 30 days, mainly due to the induction of SA dependent defense gene expressions. C6 CAGCs could protect plants from directly disrupting the microbial membranes and triggering the plant defense genes. C6 CAGC could act as a potential seed treatment agent with a dual mode of action against diverse pathogens.

## Data Availability Statement

The datasets presented in this study can be found in online repositories. The names of the repository/repositories and accession number(s) can be found in the article/Supplementary Material.

## Author Contributions

VR and AB conceived the project, designed experiments, and supervised the project. DM and KM synthesized the CA molecules. GP performed the antibacterial experiments and analyzed the data. SS performed antifungal experiments. SG helps in membrane disruption and biofilm studies. GP and VSR wrote the manuscript. VR, GJ, and AB edited the manuscript. All authors contributed to the article and approved the submitted version.

## Funding

This work was supported by RCB core grant, Ramanujan fellowship (SB/S2/RJN-046/2016) to VR, and DBT grants to AB and GJ. GP acknowledges CSIR-UGC fellowship.

## Conflict of Interest

The authors declare that the research was conducted in the absence of any commercial or financial relationships that could be construed as a potential conflict of interest.

## Publisher's Note

All claims expressed in this article are solely those of the authors and do not necessarily represent those of their affiliated organizations, or those of the publisher, the editors and the reviewers. Any product that may be evaluated in this article, or claim that may be made by its manufacturer, is not guaranteed or endorsed by the publisher.
