# Supplementary material for: Foliar Application or Seed Priming of Cholic Acid-Glycine Conjugates can Mitigate/Prevent the Rice Bacterial Leaf Blight Disease via Activating Plant Defense Genes
Source: Front Plant Sci. 2021 Sep 24;12:746912. doi: 10.3389/fpls.2021.746912 (PMC8497891; doi:10.3389/fpls.2021.746912)
Supplement: Supplementary file 1 [file Data_Sheet_1.pdf]

Supplementary information

Table S1. Primers used in this study

S.No	Primer Name	Sequence 5'-3'
	qRT_OsMKK4_F'	CCGGAGTTCAAGAGCTTCAT
	qRT_OsMKK4_R'	GACGAACCGATGTTGGAGAA
	qRT_Os ICS1_F'	TGAGTTCGCTGTTGGGATTAG
	qRT_Os ICS1_R'	ATTCCGGCACCAGCATAAA
	qRT_OsEDS1_F'	TGGTCCATGTCTGGAAGTTTAG
	qRT_OsEDS1_R'	TGATGCAGATCTGTGGTTCTC
	qRT_OsPAD4_F'	CTTCTACCAGCTCCTTGTTGAG
	qRT_OsPAD4_R'	GGCCATGGGTGATGTAAGAG
	qRT_OsNPR1_F'	CTACGAGGAAGGTTGCTGTATC
	qRT_OsNPR1_R'	TCCATCCACTTGAGCAATATCC
	qRT_PR1a_F'	GGCACTACACGCAGGTG
	qRT_PR1a_R'	CGAGTAGTTGCAGGTGATGAA
	qRT_OsVOZ2_F'	CCCGGTCCTTCTAATGCTTATC
	qRT_OsVOZ2_R'	CGCTTCTTTCGGTGGAGTAT
	qRT_SWEET14_F'	TCTCCTTCTCCCTCACCATC
	qRT_SWEET14_R'	CGTTGGGAAGAGCGACATATT
	qRT_WRKY5_F'	GCTACTACCGATGCACAAT
	qRT_WRKY5_R'	TCGTAGGTGGTGATGAGGAT
	qRT_OsWRKY13_F'	AACAGAAGGCTGTGGTTGAG
	qRT_OsWRKY13_R'	TGGTCGATCCATCCGAAGT
	qRT_OsWRKY45_F'	AACTCCAAGCACCCAAAGG
	qRT_OsWRKY45_R'	CCGATGTAGGTGACCCTGTA

Table S2. Correlation between pre and post infection of *Xoo* with response to C6 CAGC

**Correlation between pre and post infection with response to C6 CAGC**	
Colony forming units	0.9996
Evan’s blue concentration	0.8367
Lesion length	0.9862

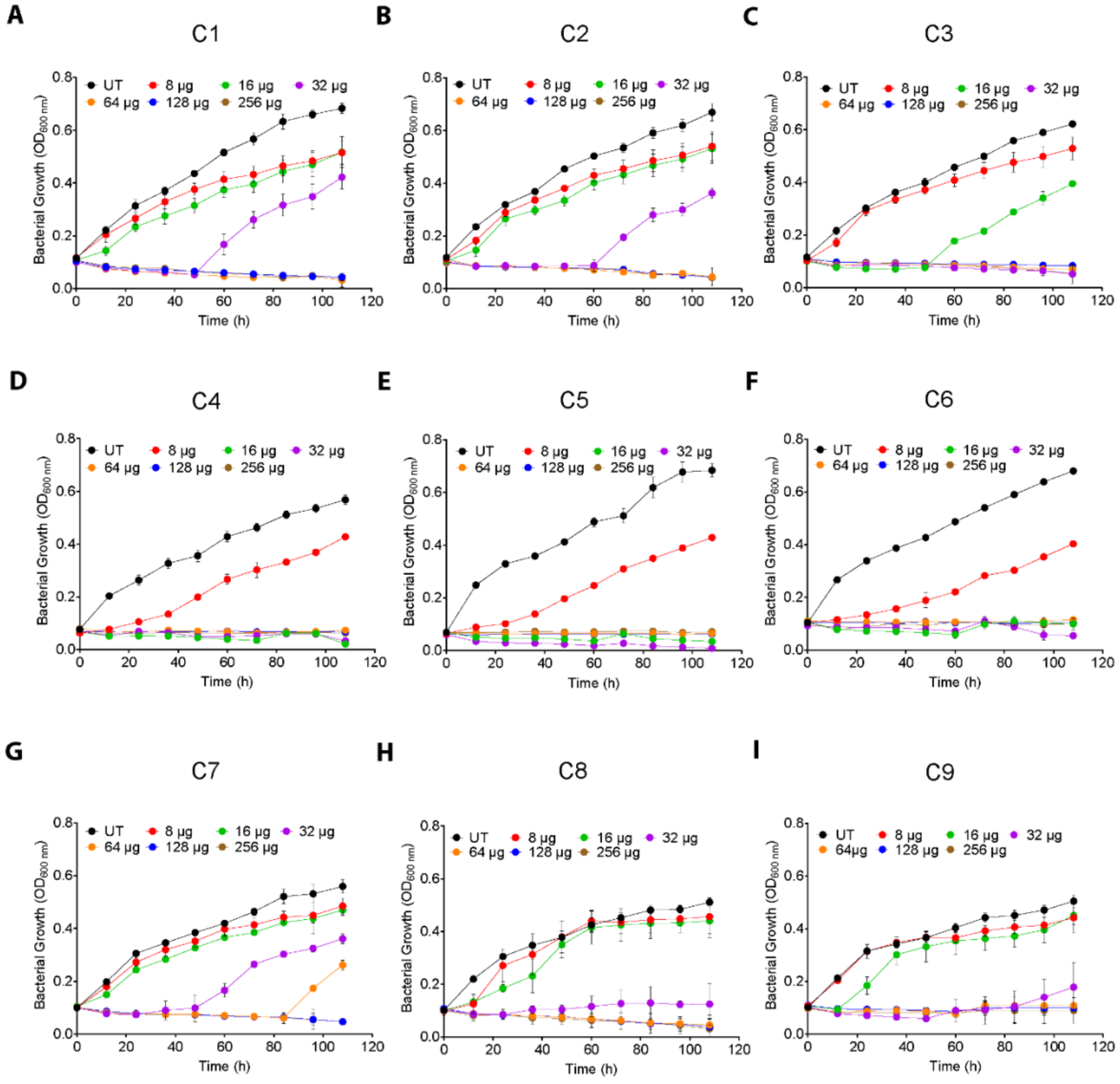

Figure S1. **Antimicrobial activity of Cholic acid glycine conjugates (CAGC).** (A-I) Screening of CAGCs library against *Xoo* in *in-vitro* growth conditions at different time points to identify potential antimicrobial compound with MIC_{99} required to kill the pathogen. *Xoo* culture of $1*10^8$ cfu/ml concentration was used as starting culture to treat with different concentrations of the CAGC molecules. The bacterial growth was measured at absorbance 600 nm at every 12 h intervals. Time-dependent growth of *Xanthomonas oryzae pv. oryzae (Xoo)* with CAGC compounds 1, 2, 3, 4, 5, 6, 7, 8, 9 and kanamycin. C4, C5 and C6 showed MIC_{99} at 16µg. The *Xoo* cultures in 96 well plates was treated with different concentrations of CAGCs in quadruplicate wells.

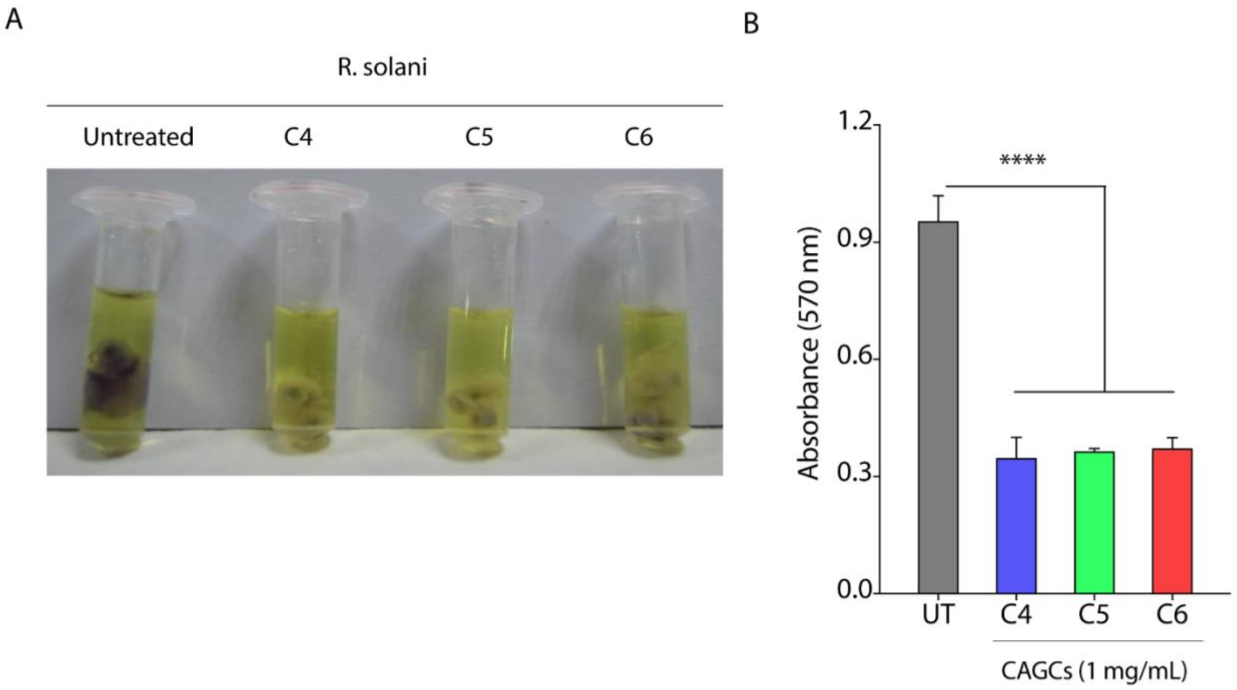

Figure S2. Response of *R. solani* mycelia with different CAGCs treatment. (A) Detection of coloured compound (formazan) produced by *R. solani* mycelia upon MTT staining. (B) Quantification of formazon by spectrophotometer analysis (OD_{570} nm). Graphs show mean values ± standard deviation (S.E). Statistical significant difference between indicated groups at $p<0.001$ (estimated by one-way ANOVA).

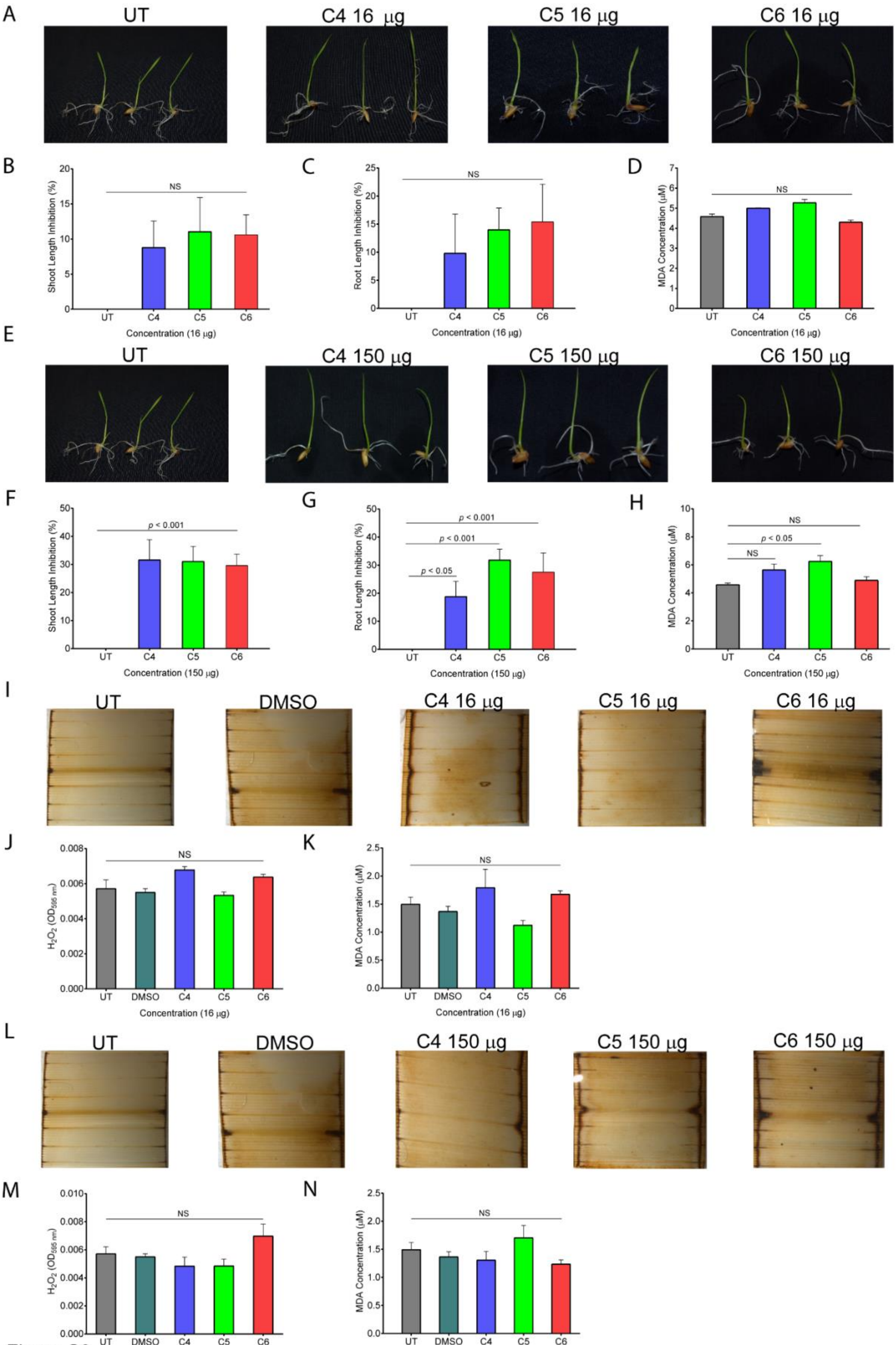
A
UT
C4 16 μg
C5 16 μg
C6 16 μg
B
Shoot Length Inhibition (%)
NS
UT C4 C5 C6
Concentration (16 μg)
C
Root Length Inhibition (%)
NS
UT C4 C5 C6
Concentration (16 μg)
D
MDA Concentration (μM)
NS
UT C4 C5 C6
Concentration (16 μg)
E
UT
C4 150 μg
C5 150 μg
C6 150 μg
F
Shoot Length Inhibition (%)
p < 0.001
UT C4 C5 C6
Concentration (150 μg)
G
Root Length Inhibition (%)
p < 0.001
p < 0.001
p < 0.05
UT C4 C5 C6
Concentration (150 μg)
H
MDA Concentration (μM)
NS
p < 0.05
NS
UT C4 C5 C6
Concentration (150 μg)
I
UT
DMSO
C4 16 μg
C5 16 μg
C6 16 μg
J
H2O2 (OD595 nm)
NS
UT DMSO C4 C5 C6
Concentration (16 μg)
K
MDA Concentration (μM)
NS
UT DMSO C4 C5 C6
Concentration (16 μg)
L
UT
DMSO
C4 150 μg
C5 150 μg
C6 150 μg
M
H2O2 (OD595 nm)
NS
UT DMSO C4 C5 C6
Concentration (150 μg)
N
MDA Concentration (μM)
NS
UT DMSO C4 C5 C6
Concentration (150 μg)

Figure S3

Figure S3: Toxicity assessment of CAGCs on rice *Taichung Native 1* (*TN1*) seedlings and leaves. Toxicity assessment in seedlings, (A & E) Rice seedling of *TN1* susceptible variety grown on 16 and 150 µg of C4, C5 and C6 treatment. Seedlings grown in distilled water are used as control. Photographs were taken after 7-days of exposure, (B & F). Inhibition of shoot length, (C & G) Root length and (D & H) Lipid peroxidation derived malondialdehyde (MDA) levels in rice seedlings. At 16µg no significant effect on the shoot length, root length inhibition (α=0.05, ****p<0.0001). Error bars indicate values of means $\pm$ SE from minimum ten biological replicates. Significant difference were determined by using One way ANOVA with Tukeys HSD test. Toxicity assessment on excised leaf disc. (A & D) The leaf discs (1 cm) from 45-day old plants of rice *TN1* variety was exposed to 16 and 150 µg concentrations of C4, C5 and C6. DMSO treated samples were used as control UT-untreated. Staining of leaf disc with DAB (B & E) Quantification of H_2O_2 content by DAB staining, formazon absorbance at 595 nm is quantified. (C & F) Levels of lipid peroxidation as quantified by MDA concentration in the leaves treated with 16 µg of C4, C5 and C6. The Leaf discs were dipped in DAB or Evan's stain for overnight and subsequently destained to remove chlorophyll with ethanol and the remaining formazon in leaves were photographed in bright field microscope. (Values are mean + SE from minimum three biological replicates). Significant difference were determined by using One way ANOVA with Tukey's HSD test. n.s-No significant difference.

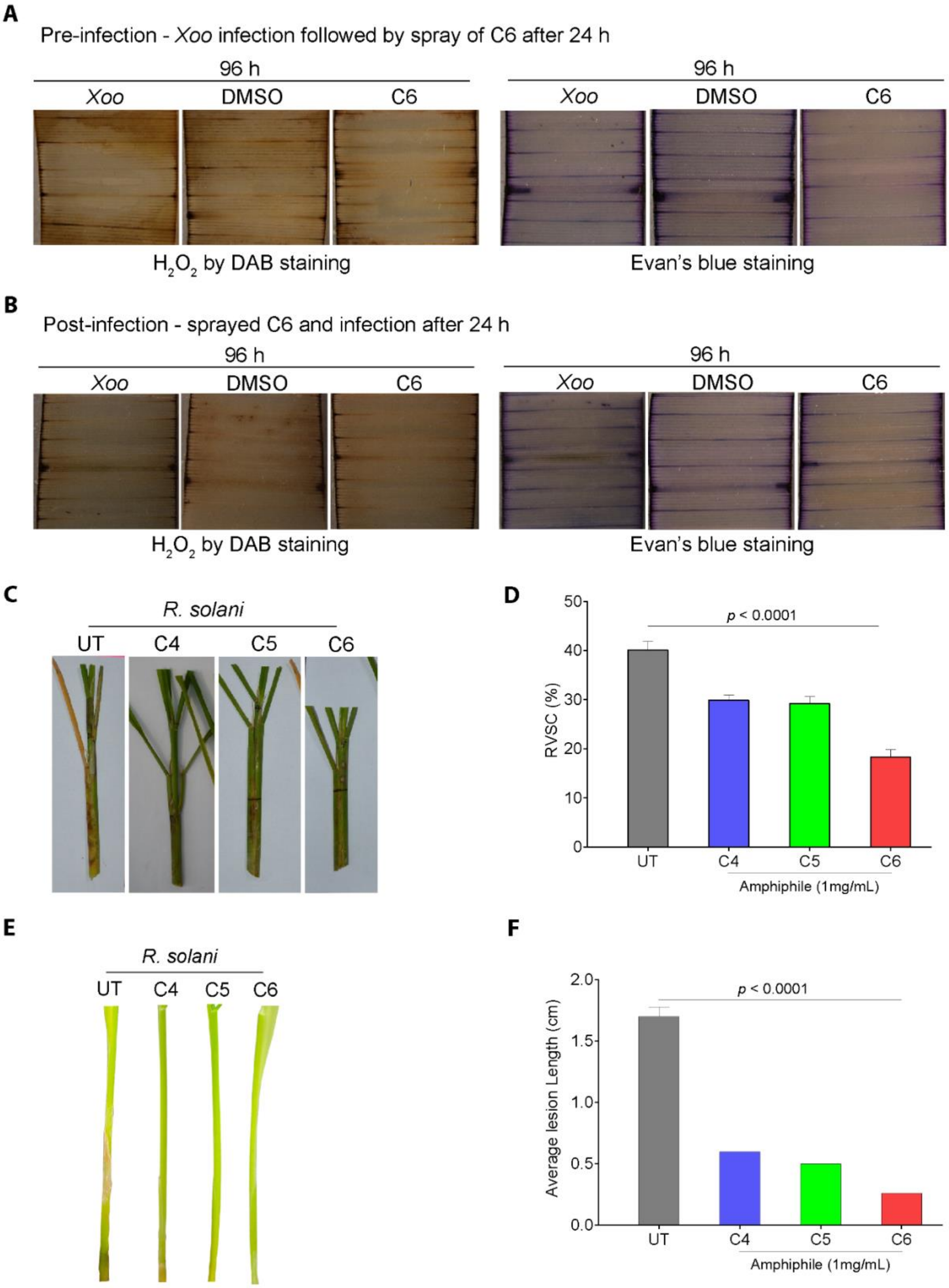

Figure S4. CAGCs provide tolerance against *Xoo* and *R. solani.* Pathogen induced H_20_2 and membrane damage in *TN1* in presence of C6. (A) Quantification of H_20_2 by DAB staining

and membrane damage by Evans blue staining from the leaf discs collected after 96 hpi of *Xoo* followed by C6 spray. (B) H_20_2 detection by DAB staining and membrane damage by Evans blue staining from Post infection of *Xoo* followed by C6 CAGC spray. The Leaf discs were dipped in DAB or Evan's stain for overnight and subsequently destained to remove chlorophill with ethanol and the remaining formazon or Evan's blue stain in leaves were photographed in bright field microscope. (C) The effect of chemical treatment on *R. solani* infection in rice. The rice tillers sprayed either with 1mg/ml solutions of chemicals (C4, C5 and C6) or distilled water (control) were subsequently infected with *R. solani*. The phenotypic assessment of disease was performed at 6 dpi. Disease symptoms in the infected rice tillers. (D) The disease severity index (Relative vertical sheath colonization - RVSC) in *R. solani* infected samples, at 6dpi. (E) Seed priming induces defence against *R. solani* infection in rice seedlings. (F) Average lesion length in primed plants after 3 dpi. The C6 amphiphile was primed to seeds and grown in pots, the fungal sclerotia was infected and observed the effect of *R. solani* infection. Graphs show mean values ± standard error (S.E). Statistical significant difference between indicated groups at $p < 0.05$ (estimated by one-way ANOVA).

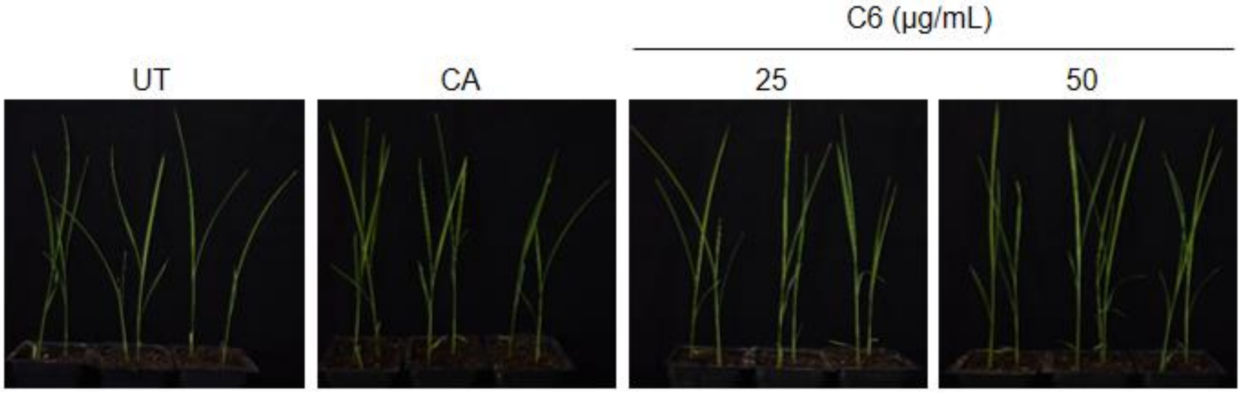

Figure S5. Growth response of CAGC primed rice seeds. Rice seeds were soaked with 25 and 50 µg/mL of C6 compound for priming. Seeds were germinated in pots, photographs were

taken after 15 days of germination showing no growth difference. UT- Unprimed seeds, CA-Cholic acid (50 µg/mL)
